# Divergent lineages in a semi‐arid mallee species, *Eucalyptus behriana*, correspond to a major geographic break in southeastern Australia

**DOI:** 10.1002/ece3.7099

**Published:** 2020-12-15

**Authors:** Patrick S. Fahey, Rachael M. Fowler, Todd G. B. McLay, Frank Udovicic, David J. Cantrill, Michael J. Bayly

**Affiliations:** ^1^ School of BioSciences The University of Melbourne Parkville Vic. Australia; ^2^ Royal Botanic Gardens Victoria South Yarra Vic. Australia

**Keywords:** climatic cycles, ddRAD‐seq, *Eucalyptus*, phylogeography, population fragmentation, species distribution model, vicariance

## Abstract

**Aim:**

To infer relationships between populations of the semi‐arid, mallee eucalypt, *Eucalyptus behriana*, to build hypotheses regarding evolution of major disjunctions in the species' distribution and to expand understanding of the biogeographical history of southeastern Australia.

**Location:**

Southeastern Australia.

**Taxon:**

*Eucalyptus behriana* (Myrtaceae, Angiospermae).

**Methods:**

We developed a large dataset of anonymous genomic loci for 97 samples from 11 populations of *E. behriana* using double digest restriction site‐associated DNA sequencing (ddRAD‐seq), to determine genetic relationships between the populations. These relationships, along with species distribution models, were used to construct hypotheses regarding environmental processes that have driven fragmentation of the species’ distribution.

**Results:**

Greatest genetic divergence was between populations on either side of the Lower Murray Basin. Populations west of the Basin showed greater genetic divergence between one another than the eastern populations. The most genetically distinct population in the east (Long Forest) was separated from others by the Great Dividing Range. A close relationship was found between the outlying northernmost population (near West Wyalong) and those in the Victorian Goldfields despite a large disjunction between them.

**Conclusions:**

Patterns of genetic variation are consistent with a history of vicariant differentiation of disjunct populations. We infer that an early disjunction to develop in the species distribution was that across the Lower Murray Basin, an important biogeographical barrier separating many dry sclerophyll plant taxa in southeastern Australia. Additionally, our results suggest that the western populations fragmented earlier than the eastern ones. Fragmentation, both west and east of the Murray Basin, is likely tied to climatic changes associated with glacial‐interglacial cycles although it remains possible that major geological events including uplift of the Mount Lofty Ranges and basalt flows in the Newer Volcanics Province also played a role.

## Graphical abstract

In this study, we investigate the phylogeography and biogeographic history of the southeast Australian mallee eucalypt, *Eucalyptus behriana*, which has a distribution divided by several major biogeographical barriers. By reconstructing the relationships between isolated populations and building species distribution models to investigate potential environmental factors influencing the species distribution, we build hypotheses regarding how the species scattered distribution comes about. Both climatic and edaphic changes are shown to likely have influenced the species distribution, with current isolated populations representing refugia where the species has survived as intervening populations have become extinct.

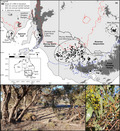

## 1 INTRODUCTION

Across the Australian continent, significant biogeographical barriers have been identified that have played a role in shaping the diversity of its unique biota (Bryant & Krosch, 2016; Schodde & Mason, 1999). These barriers range in age and porosity, leading them to influence patterns of biodiversity at many taxonomic levels; however, in this paper we focus on the infra‐specific level. In eastern Australia, phylogeographic studies have primarily been conducted on taxa of wet forests and the influence of barriers associated with intervening drier vegetation (see Bryant & Krosch, 2016), and on taxa disjunct between the Australian mainland and Tasmania (e.g., Freeman et al., 2001; Worth et al., 2017). There have been few phylogeographic studies of species from the drier vegetation that covers most of southeastern Australia, and of barriers affecting the distribution of such vegetation (e.g., French et al., 2016; Larcombe et al., 2011).

**FIGURE 1:**
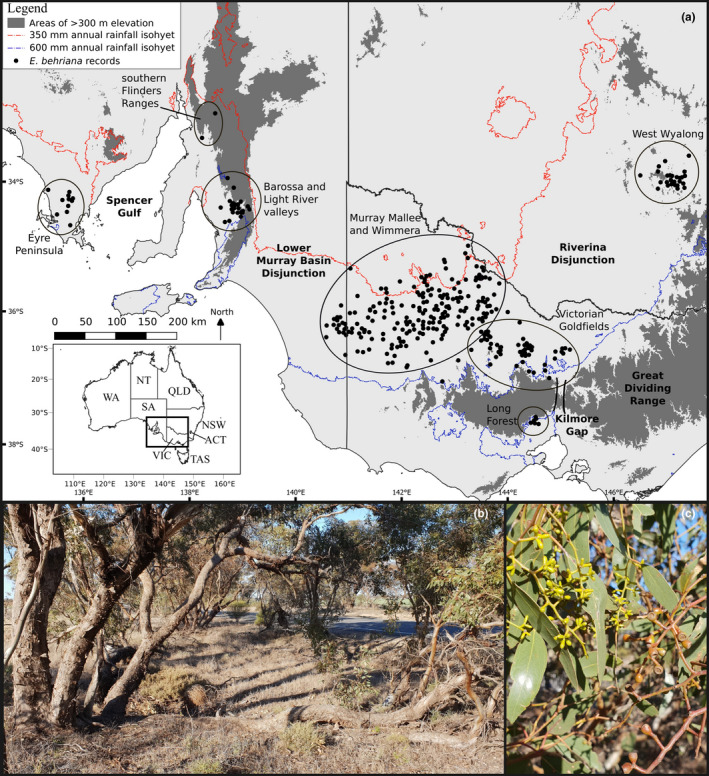
(a) Recorded sightings of *Eucalyptus behriana* downloaded from Atlas of Living Australia (2019) (filled points), grouped into geographic clusters of populations (indicated by ellipses). Areas of >300 m elevation and the 350 and 600 mm annual rainfall isohyets are shown to broadly outline the distribution of the species, while disjunctions in the species distribution are labeled as described in the text. (b) The robust mallee (multi‐stemmed) growth form typical of *E. behriana* in remanent roadside vegetation. (c) The broad, leathery, shiny leaves, and terminal clusters of small flower buds and fruit distinguish *E. behriana* from other mallee eucalypts

A prominent feature in the landscape of southeastern Australia is the Murray Basin. The lower portion of the basin encompasses the boundary between the states of South Australia, Victoria, and the southwest corner of New South Wales (Figure 1). It differs both climatically and edaphically from areas to its north‐west and southeast (Bowler et al., 2006), and has been identified as a major biogeographic barrier often referred to as the Murravian Barrier (Joseph & Omland, 2009; Schodde & Mason, 1999). The Lower Murray Basin has a complex environmental history since the late Cenozoic, with marine inundation that peaked before 7 Mya, followed by gradual marine regression (McLaren et al., 2011). Tectonic uplift ~2.4 Mya dammed the Murray River, creating a mega lake, Lake Bungunnia, covering an estimated 68,000 km^2^ of the Basin (Zhisheng et al., 1986). This starved the coastline of sediments and led to development of the Kanawinka escarpment, now forming a divide between a siliceous formation (Loxton‐Parilla Sands Strandlines) on the inland side and the calcareous Gambier Coastal Plain on the seaward side (McLaren et al., 2011). Lake Bungunnia drained within the last 700 kyr giving rise to the current channel of the Murray River and a return to depositional coastal environments (Bowler et al., 2006; McLaren et al., 2011). These processes, plus later alluvial and aeolian sediment movements, produced deep sedimentary soils and dunefields. Ongoing aridification over the last 3 Myr, overlayed with ~100 kyr glacial cycles, also affected both temperature and rainfall in the region (Mills et al., 2013).

Multiple species show a geographic disjunction across the Lower Murray Basin, but few phylogeographic studies have assessed patterns of genetic structuring associated with this disjunction. Examples of plant phylogeographic studies include those of *Hardenbergia violacea* (Schneev.) Stearn (Larcombe et al., 2011), *Eucalyptus globulus* (Jones et al., 2013), and the genus *Correa* (French et al., 2016). All of these highlight a deep genetic divergence associated with the basin, which could be associated with history of vicariant separation of populations on either side, although in *Correa* subsequent reconnection of populations across the Murray Basin was also inferred. Genetic studies of additional taxa with disjunct distributions across the Lower Murray Basin could assess whether common genetic patterns can be identified in the history of the vegetation of this region, associated with either vicariance, where disjunct species predate formation of the barrier, or dispersal subsequent to formation of the barrier.

*Eucalyptus behriana* F. Muell., a mallee (lignotuberous, multistemmed shrub) in *Eucalyptus* section *Adnataria* (Boxes and Ironbarks), is a species that occurs both to the west and in the eastern half of the Murray Basin (Figure 1) (Brooker et al., 2015). It occurs mainly as a member of tall mallee woodlands in areas of 350–600 mm annual rainfall with shallow soils (Benson, 2008; Myers et al., 1986; Nicolle, 2014). The distribution of the species shows several other large disjunctions, making its biogeographical history potentially informative regarding the broader environmental evolution of southeastern Australia, and a good test case for whether there are recurring patterns of genetic relationships in dry sclerophyll plant taxa across the Murray Basin.

The distribution of *E. behriana* includes at least three isolated populations on the north‐west side of the Murray Basin: a population on the lower Eyre Peninsula, another in the Barossa Valley and Light River Valley, and a third, smaller population in the southern Flinders Ranges (~20 individuals) (Figure 1) (Atlas of Living Australia, 2019). Single herbarium specimens represent two additional populations in the southern Flinders Ranges (Nicolle, 2014); however, those populations were not observed in the course of this study and their continued survival is uncertain. The largest area of *E*. *behriana* populations occurs within the eastern Murray Basin in the Wimmera and southern Murray Mallee bioregions (sensu Interim Biogeographic Regionalisation for Australia version 7 (IBRA7), Australian Government, 2017). Within these regions, *E. behriana* mainly occurs in depressions with heavier soils and higher water holding capacity than surrounding sand plains (Nicolle, 2014; White, 2006), and it is rare in the adjoining Lowan Mallee bioregion dominated by younger aeolian dunes (Conn, 1993). Further east, sporadic populations occur on Ordovician and Devonian sandstone outcrops in the Victorian Goldfields (Raymond et al., 2012). Other isolated populations occur at Long Forest, the only area of mallee vegetation south of the Great Dividing Range (GDR), and around the town of West Wyalong several hundred kilometers north in the South‐West Slopes region of New South Wales (Benson, 2008). At Long Forest, the species occurs on solodic soils developed on an outcrop of Ordovician sediments, largely surrounded by richer soils on significantly younger basaltic volcanic flows (Myers et al., 1986).

Our aim in the current study was to assess patterns of genetic variation in *E. behriana* to build hypotheses regarding evolution of major disjunctions in the species' distribution and to expand understanding of the biogeographic history of southeastern Australia. Specifically, we sought to assess the significance of the Lower Murray Basin as a barrier to gene flow, relative to the other geographic disjunctions in *E. behriana*, to contribute to understanding of the importance of this feature in the history of the flora. We also sought to assess whether genetic patterns showed any support for recent dispersal, over a null hypothesis of vicariance, in the history of the species. To achieve these aims, we analyzed anonymous nuclear genome loci generated through double digest restriction site‐associated DNA sequencing (ddRAD‐seq) and built species distribution models (SDMs).

## 2 METHODS

### 2.1 Sampling

**FIGURE 2:**
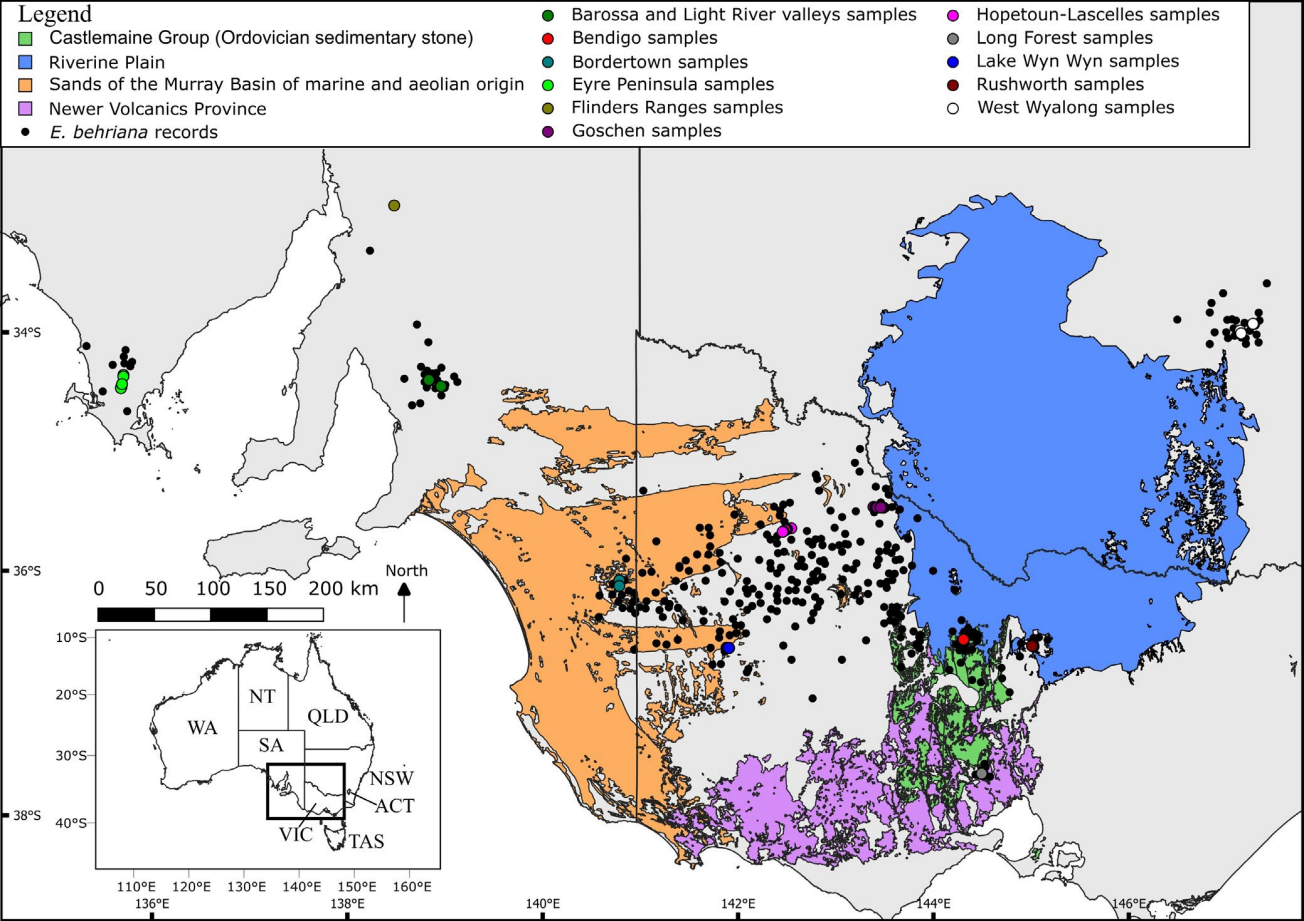
Recorded sightings of *Eucalyptus behriana* downloaded from Atlas of Living Australia (2019) (black points) and sample collection locations for this study (colored points). The area of sandy soils of the lower Murray Basin is shown in orange, the alluvial deposits of the Riverine Plain in blue, Newer Volcanics province in purple and the Castlemaine Group in green

Populations were sampled across the range of *E. behriana* (Figure 2). We sampled 1–11 individuals from each sampling location, depending on population size (Table 1). Samples were collected ~100 m apart to minimize the chances of sampling siblings, as most eucalypt seeds fall within 20 m of parent trees (Booth, 2017). Leaves showing minimal signs of disease or damage were collected into coffee filters and silica desiccating beads, and at least one voucher herbarium specimen was taken per sampling location (Table 1).

**TABLE 1:** Collection and accession details of samples of *Eucalyptus behriana* used in this project

Population	Number of samples in final dataset	Approximate latitude	Approximate longitude	Herbarium specimen
Barossa and Light River valleys	10	−34.4256	138.8892	MELUD122586a
Bendigo	10	−36.5784	144.3117	MELUD122574a
Bordertown	7	−36.0901	140.7878	MELUD122623a
Eyre Peninsula	9	−34.4431	135.6940	MELUD122609a, MELUD122610a
Flinders Ranges	1	−32.9157	138.4834	MELUD122621a
Goschen	10	−35.4725	143.4296	MELUD122640a, MELUD122641a
Hopetoun‐Lascelles	10	−35.6608	142.4926	MELUD122639a
Lake Wyn Wyn	10	−36.6359	141.8997	MELUD122626a, MELUD122627a
Long Forest	11	−37.6635	144.5026	MELUD122643a
Rushworth	10	−36.6151	145.0204	MELUD122577a
West Wyalong	9	−33.9635	147.2141	MELUD122648a

Note

Coordinates represent the approximate centre of the collecting area for each population.

### 2.2 DNA extraction and library preparation

The modified CTAB protocol of Schuster et al. (2018) was employed for DNA isolation from 70–80 mg of dry leaf material for 99 samples. Extraction purity was checked on a Nanodrop 2000 spectrophotometer (Thermo Fisher) and quantified using a Qubit 2.0 fluorometer (Thermo Fisher). DNA was standardized to a concentration of 100 ng/μl in a total volume of 15 μl with purified water. ddRAD libraries were prepared following Peterson et al. (2012) and Yang et al. (2016), except that inline barcodes were not used in the adaptor sequences. EcoRI‐HF and MspI restriction enzymes were used to digest DNA, which Yang et al. (2016) showed produced a large number of 400–700 base pair fragments from the *E. grandis* genome in silico. To increase nucleotide diversity for sequencing on a NextSeq platform, eight Illumina adaptors with varying extra bases included at the 5ʹ end, four compatible with each overhang left by the restriction enzymes, were mixed in equal concentrations and ligated to the samples. Serapure SPRI beads (Rohland & Reich, 2012) were used to remove reagents and DNA fragments <300 bp in length. Libraries were amplified by qPCR using indexing primers for multiplexing, and samples were pooled based on end‐point fluorescence. Size selection of the library used a Pippin Prep and 1.5% agarose Marker K cassette following the manufacturer's protocol (Sage Science Inc, 2012) with a tight selection around a target length of 750 bp. Final library quantification was performed on a 2,200 Tape Station (Agilent) using a D1000 kit. The library was sequenced on an Illumina NextSeq 500 using a 300 cycle, 2× paired‐end reads kit at the Walter and Eliza Hall Institute of Medical Research (WEHI) Genomics Hub, Melbourne, Australia.

### 2.3 Read trimming, quality control, and data assembly

Reads were paired in Geneious v11.1.5 (https://www.geneious.com, Kearse et al., 2012) and trimmed to remove restriction site residues and adaptor diversity bases using the Cutadapt 2.8 python script (Martin, 2011) with a maximum error rate of 0.5. Paired and trimmed reads from the 97 samples successfully sequenced were put through the ipyrad pipeline (v.0.9.33) (Eaton & Overcast, 2020) to assemble loci (see Table S1.1 available on Dryad at https://doi.org/10.5061/dryad.v9s4mw6sm for parameters used). Data were treated as paired‐end ddRAD data and assembled using the reference option, mapping to the eleven chromosomes of the *Eucalyptus grandis* genome (Myburg et al., 2014). We targeted only loci from the nuclear genome to avoid any potential confounding of results due to the well‐established cytonuclear discrepancy in the eucalypts (Alwadani et al., 2019; Nevill, Després, et al., 2014). Multiple values for several ipyrad parameters were tested to find the most informative dataset possible. The analysis using a 0.85 clustering threshold, 6 read minimal coverage, and a minimum of 75% of samples represented at each locus was identified as the optimized dataset, as it contained as much data as possible while still allowing for confidence in the base calls and analyses not being computationally limited.

### 2.4 Analyses

The program VCFtools (Danecek et al., 2011) was used to construct a SNP dataset that included only one SNP per 5,000 bp window of the *E. grandis* reference genome allowing no more than 25% missing data at each site, giving a final dataset size of 4,365 SNPs. An average of 2.4% of alleles present in each population was private to that population and these were maintained in the dataset. Population variation statistics, expected heterozygosity and mean *F*_ST_ values with confidence intervals based upon 100 bootstrap replicates over loci, were calculated on this SNP dataset in the R statistical environment (R Development Core Team, 2016) using the *adegenet* (Jombart & Ahmed, 2011) and *hierfstat* (Goudet, 2005) packages. Additionally, a Mantel test for isolation by distance (IBD) was performed using the *mantel* function of the *vegan* package (Oksanen et al., 2019). To investigate patterns of reticulation and uncertainty, a phylogenetic network (NeighbourNet) was built from the concatenated alignment generated by ipyrad using uncorrected P distances and the median network algorithm under default parameters in SplitsTree v4.15.1 (Huson & Bryant, 2006).

### 2.5 Species distribution modeling

Maxent (Phillips et al., 2006) was used to build species distribution models at a cell scale of 2.5 arc‐minutes. Environmental variables used included the 19 bioclimatic variables from WorldClim 2 (Fick & Hijmans, 2017) and five soil parameters from the Soil and Landscape Grid of Australia (Viscarra Rossel et al., 2015), measured at a depth of 30–60 cm where applicable: clay percent content, sand percent content, soil depth, soil water holding capacity and soil pH. As historic soil data are not available, we built two models, one limited to climatic variables which was projected on glacial maximum (~20 kya) climate variables from WorldClim 1.4 (Hijmans et al., 2005), and one for current conditions only that included climatic and soil variables. The MaxentVariableSelection package for R (Jueterbock et al., 2016) was used to incrementally reduce the number of variables while testing regularization factors between 0.5 and 15 in 0.5 increments, and the Akaike information criterion was used to choose optimal models. For the climate only model, the optimized model included four predictors (annual mean temperature, mean temperature of wettest quarter, annual precipitation, precipitation of warmest quarter) and regularization parameter of 1.5; the soil and climate model included five predictors (annual mean temperature, mean temperature of wettest quarter, annual precipitation, soil clay content, soil pH) and a regularization parameter of 1.

## 3 RESULTS

### 3.1 Data

The final dataset included 97 samples and 5,224 loci, with 181,049 variable sites, of which 95,246 were parsimony informative. The number of assembled loci was uneven between individual samples, ranging from 2,405 to 5,018 leading to 23.6% missing data in the final dataset; however, the reconstruction of biogeographically sensible patterns in downstream analyses suggests that missing data did not meaningfully influence relationships between samples. Datasets used in analyses are available on Dryad at https://doi.org/10.5061/dryad.v9s4mw6sm.

### 3.2 Population variation

Observed heterozygosity of individuals ranged from 0.0107 to 0.0141 (see Table S1.2 available on Dryad at https://doi.org/10.5061/dryad.v9s4mw6sm) and population expected heterozygosity (Table 2) was consistent across populations (*H*_e_ = 0.0685–0.0815). Interpopulation *F*_ST_ values were low (mean 0.0663, standard deviation 0.0364); however, no confidence intervals overlapped with zero. Highest average *F*_ST_ values were observed between the two larger western populations and all other populations, include between these two populations (Table 2). All pairwise values between sites in the Goldfields, Wimmera and Murray Mallee were below the overall average at <0.05, with the only pairing of populations east of the Lower Murray Basin with an above‐average pairwise *F*_ST_ being the two outlying populations at Long Forest and West Wyalong. The Mantel test for isolation by distance showed strong support for moderate IBD (*p* > .001, *r* = .5107).

**TABLE 2:** *Eucalyptus behriana* population expected heterozygosity and interpopulation *F*_ST_ values calculated from ddRAD‐seq data

Population	*H*_e_	Interpopulation *F*_ST_ value											
		Eyre Peninsula	Barossa and Light River valleys	Flinders Ranges	Bordertown	Lake Wyn Wyn	Hopetoun‐Lascelles	Goschen	Bendigo	Rushworth	Long Forest	West Wyalong
Eyre Peninsula	0.0696		0.0950–0.1222	0.1071–0.1829	0.0979–0.1253	0.0974–0.1279	0.0959–0.1194	0.0978–0.1235	0.1034–0.1298	0.1137–0.1427	0.1350–0.1658	0.1105–0.1404
Barossa and Light River valleys	0.0815	0.1081		0.0161–0.0767	0.0578–0.0822	0.0658–0.0867	0.0609–0.0829	0.0649–0.0897	0.0708–0.0890	0.0828–0.1085	0.0975–0.1262	0.0760–0.0966
Flinders Ranges	—	0.1505	0.0468		0.0198–0.0925	0.0437–0.1036	0.0296–0.0886	0.0145–0.0752	0.0451–0.1027	0.0621–0.1291	0.0971–0.1630	0.0645–0.1333
Bordertown	0.0747	0.1101	0.07	0.0564		0.0223–0.0436	0.0182–0.0361	0.0101–0.0278	0.0249–0.0414	0.0335–0.0560	0.0462–0.0736	0.0412–0.0616
Lake Wyn Wyn	0.0752	0.111	0.0762	0.0756	0.0333		0.0180–0.0309	0.0141–0.0285	0.0199–0.0335	0.0241–0.0433	0.0480–0.0696	0.0412–0.0618
Hopetoun‐Lascelles	0.0786	0.1049	0.0728	0.0576	0.0265	0.024		0.0056–0.0174	0.0147–0.0309	0.0254–0.0433	0.0409–0.0620	0.0314–0.0492
Goschen	0.0799	0.1111	0.0778	0.0474	0.0199	0.0209	0.0107		0.0185–0.0332	0.0318–0.0498	0.0463–0.0683	0.0312–0.0494
Bendigo	0.0744	0.1145	0.0806	0.0769	0.034	0.0273	0.023	0.0258		0.0090–0.0192	0.0378–0.0556	0.0259–0.0426
Rushworth	0.0735	0.1282	0.0971	0.1019	0.0436	0.0341	0.0337	0.0416	0.0144		0.0419–0.0641	0.0333–0.0505
Long Forest	0.0685	0.1499	0.1135	0.1331	0.0581	0.058	0.0508	0.0564	0.046	0.0542		0.0650–0.0882
West Wyalong	0.0722	0.1237	0.0867	0.0969	0.0527	0.0515	0.0411	0.0408	0.034	0.0405	0.0743	

Note

*F*_ST_ values below the diagonal are the average values and those above the diagonal are 95% confidence intervals based upon 100 bootstrapping replicates. Darker red cells indicate a higher than average *F*_ST_ value and therefore greater divergence, while darker blue cells represent below mean *F*_ST_ values and lower population divergence. No *H*_e_ value was calculated for the Flinders Ranges population as only one sample was included in the dataset.

### 3.3 NeighbourNet network

**FIGURE 3:**
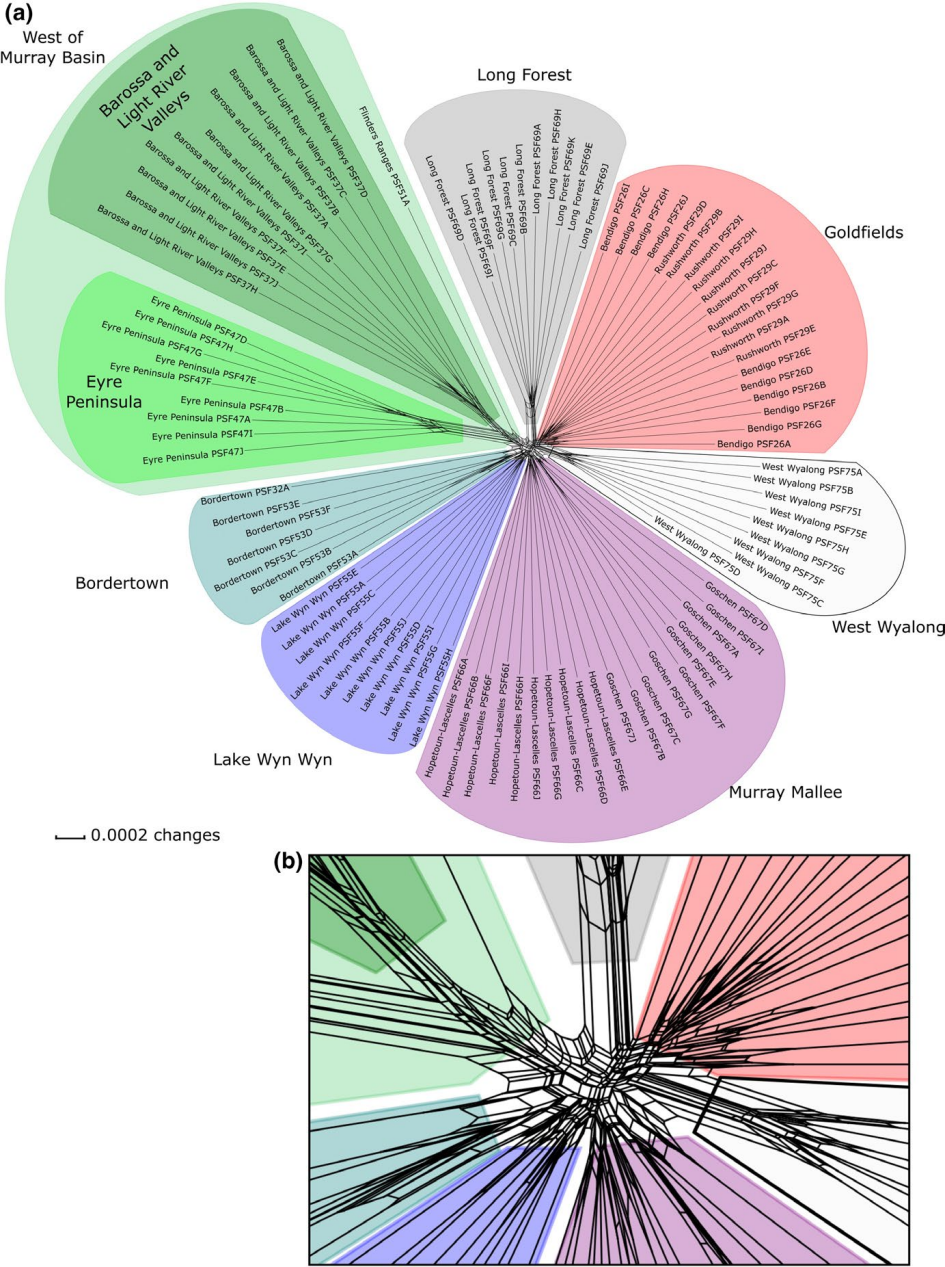
SplitsTree network of ddRAD‐seq alignment for *Eucalyptus behriana*. Major groupings are represented with colored clouds. An eastwest split exists between the major groups and outlying populations formed separate clusters; however, the sampling sites in both the Goldfields, and the Murray Mallee did not form discrete clusters. Plot (a) shows the entire network, with (b) showing the detail of relationships at the core of the network

The NeighbourNet network (Figure 3) showed the western populations (Eyre Peninsula, Barossa and Light River valleys, and Flinders Ranges) as a grouping, albeit with greater between‐population divergence than eastern populations. Among western populations, the single sample from the Flinders Ranges was placed as sister to the two larger populations. The most distinct eastern populations were the geographically isolated Long Forest and West Wyalong populations, with the two goldfields populations forming a single cluster, as did the two sampling populations from the Murray Mallee (Hopetoun‐Lascelles and Goschen), which grouped close to those from Bordertown and Lake Wyn Wyn. The West Wyalong population clustered closest to the Goldfields populations; with the Long Forest population clustering nearest this West Wyalong + Goldfields grouping.

### 3.4 Species distribution models

**FIGURE 4:**
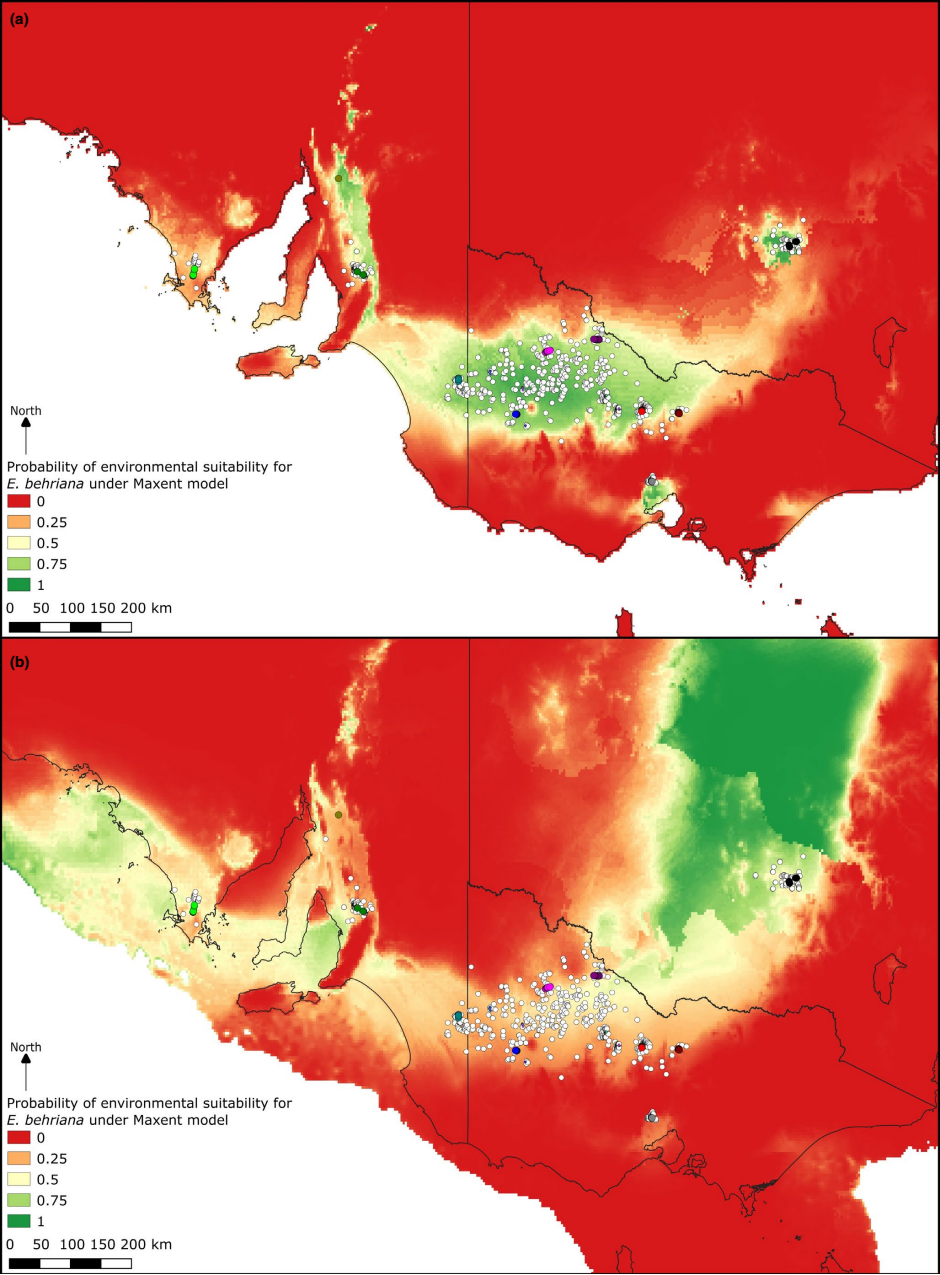
Optimized Maxent model predictions of environmental suitability under (a) current climatic conditions, and (b) projected onto reconstructed LGM climate. This optimized model used a regularization factor of 1.5 and four predictor variables: mean annual temperature, mean temperature of wettest quarter, mean annual precipitation, and mean precipitation of warmest quarter. Point records include both those downloaded from the Atlas of Living Australia and collections used in this study as per Figure 1

**FIGURE 5:**
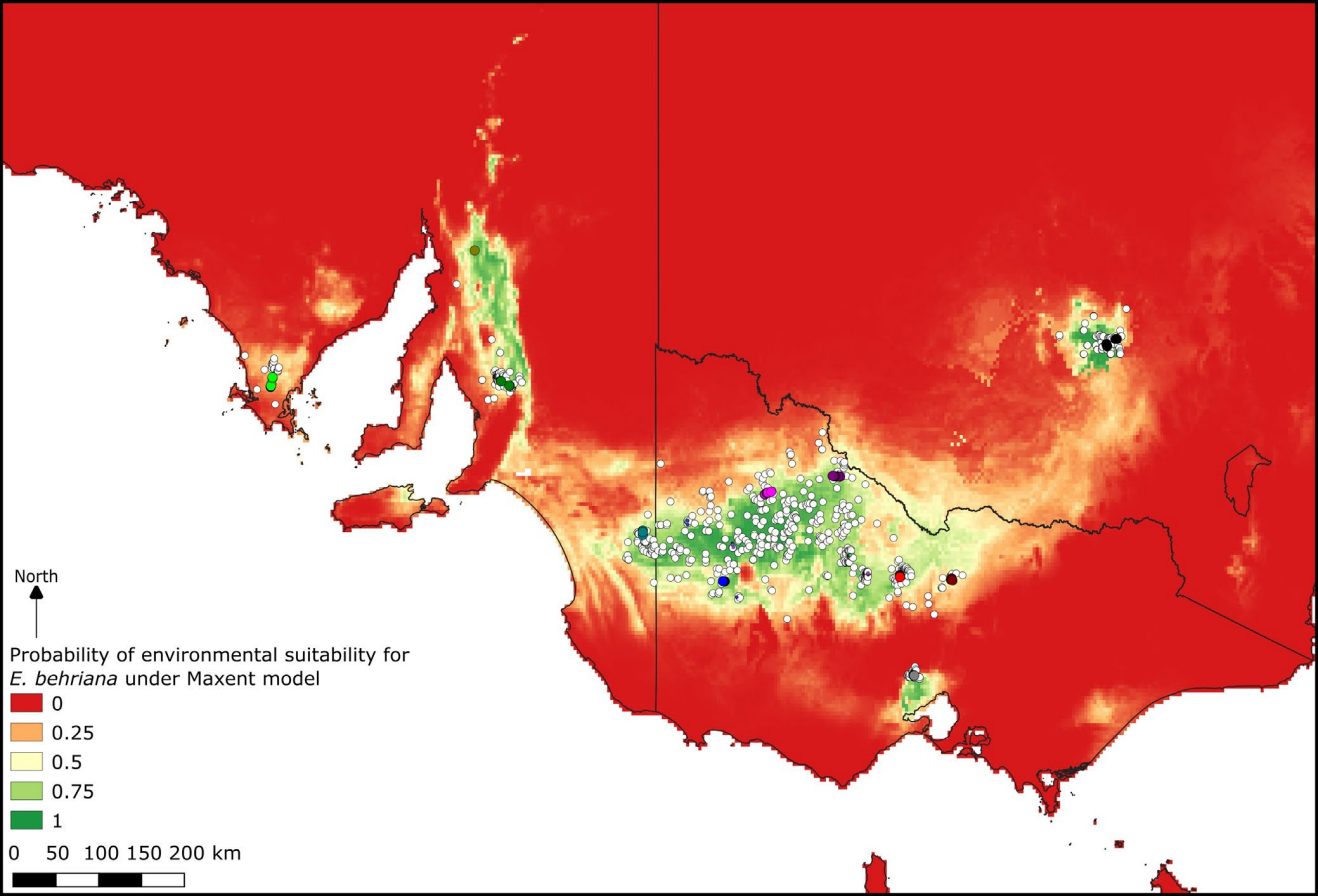
Optimized Maxent model predictions of environmental suitability under current climatic and edaphic conditions. This optimized model used a regularization factor of 1 and five predictor variables: mean annual temperature, mean temperature of wettest quarter, mean annual precipitation, soil clay content (30–60 cm below soil surface), and soil pH (30–60 cm below soil surface). Point records include both those downloaded from the Atlas of Living Australia and collections used in this study as per Figure 1

The species distribution models (Figures 4 and 5) show a good fit to the current distribution of the species, although the soil and climate model (Figure 5) was a tighter fit to the species distribution in the Murray Basin and inland slopes of the GDR, suggesting that soil factors indeed contribute to these disjunctions. When the climate only model was projected onto modeled last glacial maximum climatic conditions (Figure 4b), the areas of potentially suitable environment moved far to the north, with low suitability across the areas the species currently occupies.

## 4 DISCUSSION

### 4.1 *Eucalyptus behriana* population variation

We have shown that *E. behriana* has genetic diversity, as measured by heterozygosity, consistent with what has been reported in other box eucalypts (Jordan et al., 2016; Murray et al., 2019; Supple et al., 2018) and only moderate genetic structuring between populations. Despite this, the species‐wide *F*_ST_ (0.066) was the highest so far reported for any species in *Eucalyptus* section *Adnataria* (0.017–0.018 in *E. albens* and *E. sideroxylon* (Murray et al., 2019), 0.04 in *E. melliodora* (Supple et al., 2018), 0.02 across the *E. brownii*/*E. populnea* cline (Holman et al., 2003)), indicating greater, albeit still low, genetic differentiation between populations. Relative to these species, which have near continuous distributions, stronger population structuring is to be expected in *E. behriana*, with the fragmented nature of its distribution likely reducing gene flow between populations. The only previously reported *F*_ST_ value for a eucalypt species higher than that of *E. behriana* was from *E. globulus* sensu lato (*F*_ST_ = 0.08) (Jones et al., 2002), which is morphologically variable across a disjunct distribution and is recognized as four distinct species by Nicolle (2018). Overall, the highest population level divergences were observed between populations separated by the Lower Murray Basin (Table 2); divergence between the two largest western populations, separated by the Spencer Gulf, was also high.

The observed support for isolation by distance in *E. behriana* is unsurprising given the outlying populations at extremes of the distribution, which are likely isolated from gene flow with other populations, and it does not rule out the existence of discrete population structuring. Similar patterns are seen in other widespread tree species with large population sizes across various regions of the world, including other *Eucalyptus* species (Bloomfield et al., 2011; Jones et al., 2013; Murray et al., 2019; Nevill, Bradbury, et al., 2014; Supple et al., 2018), *Quercus* (Ju et al., 2019), *Populus* (Keller et al., 2010), and *Pinus* (Potter et al., 2015), which show limited genetic structuring; with isolation by distance explaining much of the genetic divergence. In the case of eucalypts, it has been suggested that their high recombination rates (Gion et al., 2016), preferential outcrossing (Byrne, 2008; Horsley & Johnson, 2007), and substantial inbreeding depression in the case of selfing (Nickolas et al., 2019), all favor the retention of genetic diversity and a lack of geographical structuring despite lacking long distance dispersal capabilities (Booth, 2017).

### 4.2 Mechanisms and timing of population disjunctions

Mallee eucalypts, such as *E. behriana*, typically have long life spans, associated with a large lignotuber rich in dormant buds and energy reserves that provide substantial capacity to recover from fires and droughts (Noble, 2001). They also have low recruitment rates (Wellington & Noble, 1985) and limited capacity for seed dispersal (Booth, 2017). Given these traits and the lack of evidence for recent admixture between disjunct populations in the phylogenetic network and *F*_ST_ values, we suggest that it is more parsimonious that disjunctions in the distribution of *E*. *behriana* result from fragmentation of a once more contiguous distribution, rather than long distance seed dispersal between areas, although the latter cannot be ruled out. We have not attempted to put an absolute age on lineage divergences in this study, as there is a lack of closely related fossils for calibrating analyses, and the most recent dated phylogeny of the eucalypts failed to resolve tip relationships at the sectional level, including within *Adnataria* (Thornhill et al., 2019), limiting options for secondary calibrations. Instead, we have considered relative levels of genetic divergence between populations as a means of reconstructing the order of population isolation and by combining this with knowledge of historical environmental change developed hypotheses regarding the species biogeographical history.

### 4.3 East‐west divergence across the Murray Basin

We found a substantial genetic divide between the populations to the east and west of the Murray Basin (Figure 3); however, without an outgroup, we are unable to determine with certainty whether this reflects monophyletic groups, or one clade nested within the other. If the latter is true, based upon the level of genetic diversity, we suggest there is a stronger case for the eastern clade being nested within the west than the reverse. The large differentiation between the populations in the west suggests that fragmentation occurred in these western populations earlier and more completely than in the currently larger eastern populations. These results are largely congruent with patterns in *Hardenbergia violacea* (Larcombe et al., 2011) and *Correa* spp. (French et al., 2016), adding weight to the hypothesis that this deep east‐west split in the Murray Basin may be a recurring pattern among dry sclerophyll plant taxa, reflecting the historical environmental change in the region.

### 4.4 Diversity of western populations

Our findings suggest that the western populations may not have been the small and geographically restricted populations they are today for an extended period, as genetic diversity in these populations would have been reduced by genetic drift and bottlenecking if this were the case (Amos & Harwood, 1998). Indeed, we see the opposite to this with the highest genetic diversity being observed in these currently small, restricted western populations. While the species was likely not significantly more widespread prior to European land clearing, the complete extent of the preclearing populations is unknown, with the Maxent model employing both soil and climatic variables suggesting minimal areas of suitable environments on the Eyre Peninsula, but large areas of high suitability between the Barossa Valley and southern Flinders Ranges (Figure 5). In the Barossa and Light River valleys *E*. *behriana* is the dominant tree in the communities in which it occurs; however, these communities only survive as remnant roadside vegetation in an extensively cleared landscape (Neagle, 2008). The Eyre Peninsula population has also been affected by land clearing but covers a narrower geographic area and *E*. *behriana* is not the dominant species where it occurs, rather occurring only along drainage lines, aside from a poorly drained depression west of Cummins where it is co‐dominant with *E. odorata* (Smith, 1963). There is no information on the history of the Flinders Ranges populations, so it is impossible to say to what extent natural versus human factors have contributed to their highly limited extent, however the sample included in this study is genetically distinct from all other populations, potentially being sister to the other two western populations, suggesting either extended isolation or small population size. The small size and high genetic distinctiveness of these western populations highlights that they should be considered of conservation importance.

This pattern in the western clade of genetically diverse, geographically restricted populations could be interpreted as arising from a population retraction to what are effectively current refugia in response to environmental change. An environmental factor that may have driven this retreat is the glacial cycles of the Pleistocene. While the term refugia is most commonly used to refer to areas of constant occupancy by a taxon through glacial periods, it stands to reason that those species that replace the retreating taxa where glaciation does not occur may retreat to refugial areas under interglacial conditions (Bennett & Provan, 2008). This is particularly likely in the case of Australia as there was not large‐scale formation of icesheets across the landscape, rather widespread cooling and drying of the climate (Barrows et al., 2001). Here we put forward the hypothesis that the western populations may have been initially fragmented by a climatic shift associated with glaciation cycles with the higher rainfall of interglacial conditions allowing taller eucalypt species to replace *E. behriana* in areas between the current populations. The associated sea‐level rise also likely played a role, with the Spencer Gulf that sits between the Eyre Peninsula, and Barossa and Light River valleys populations being above sea‐level during glacial climates, thus allowing population connectivity across the region, a hypothesis supported in our SDM which show high environmental suitability under glacial climates in the Spencer Gulf. A second hypothesis regarding this fragmentation is that it has been a gradual process associated with the long‐term climatic trends that operate on time spans longer than recent glacial cycles. The Australian climate has been gradually drying over the last ~10 MY, although a wetter peak is known from ~5–3 Mya (Byrne et al., 2008), and it is entirely possible that this drying has caused the loss of *E. behriana* populations from previously occupied areas across what is today the north of the Spencer Gulf and upper Eyre Peninsula. These areas receive <350 mm of annual rainfall under the current climate, the lower bounds of the realized niche of *E. behriana*, however there are large areas of 350–500 mm annual rainfall on the Eyre Peninsula and coastal side of the Mount Lofty Ranges where *E. behriana* does not occur, suggesting climate alone cannot explain the current distribution of the species west of the Murray Basin. It therefore seems likely that the edaphic conditions where the species currently occurs play a role in facilitating persistence, which is congruent with the finding of a better Maxent model when edaphic predictors were included (Figure 5). Additionally, uplift of the Mount Lofty Ranges continues to the present (Tokarev, 2005), and therefore changes in topography may be one of the factors that contributed to establishing the disjunctions in the taxon's western populations.

### 4.5 History of the West Wyalong population

The low level of genetic distance between the Goldfields and West Wyalong populations suggests that these populations diverged comparatively recently relative to other eastern populations (Figure 3), despite the large geographic separation of these populations. Though there are no established biogeographical barriers in the literature that match this disjunction, it corresponds to the riverine plain of the Murray, Murrumbidgee and Lachlan rivers. This is a largely treeless region of deep alluvial soils (Eardley, 1999), an edaphic environment that *E. behriana* is not known to inhabit, and that the SDM including edaphic factors (Figure 5) returns as lower environmental suitability than the climate only SDM (Figure 4a). It is therefore suggested that these soils form the basis of this barrier. Investigation of collecting records of plant taxa that occur in the vicinity of both the Victorian Goldfields and West Wyalong on the Atlas of Living Australia (Atlas of Living Australia, 2019) identified a number of other species with similar disjunctions, including *Eucalyptus polybractea* R.T.Baker, *E. viridis* R.T.Baker, and *Melaleuca uncinata* R.Br. Several other species have distributions that follow the western arc of the GDR between these locations but are absent from the riverine plain, including *Pultenaea largiflorens* F.Muell. ex Benth., *Hibbertia crinita* Toelken, and *Acacia montana* Benth. In the case of *E. behriana*, we infer historical connection between these two areas was probably of a similar nature, following the inland slopes of the ranges (consistent with our SDMs under current environmental conditions) and that intervening populations have been lost, presumably because of subsequent environmental changes.

### 4.6 History of Long Forest population

We found only weak evidence for the relationship between the Long Forest population and other eastern populations, although the NeighbourNet network (Figure 3) suggests a potential relationship between this population and the cluster consisting of the Goldfields and West Wyalong population. The Long Forest population is unique in representing the only area of mallee vegetation south of the GDR and has previously been studied in an ecological context by Myers et al. (1986). In their study, these authors put forward the hypothesis that Long Forest was colonized by *E. behriana* through the Kilmore Gap (Figure 2), a low saddle in the GDR to the north‐west of Long Forest, possibly under the climate of a glacial maximum, which would fit with the NeighbourNet results. Under this hypothesis, the Long Forest represents a refugium where the species has persisted, due to local edaphic conditions (Myers et al., 1986), as a shift to an interglacial climate led to expansion of taller woodland and forest communities at the expense of mallee vegetation. Another option we cannot rule out, but still congruent with the glacial cycle driven changes to distribution, is that the historic connection of the Long Forest population to others of the species was not through the Kilmore Gap, but around the southwest of the GDR. The SDMs (Figures 4a and 5) for current conditions show a low probability of occurrence through both these routes, with the link from Long Forest to the Wimmera being returned as slightly higher probability of environmental suitability. However the probability of suitability of both the Kilmore Gap and the western route was lower in the LGM model (Figure 4b) than both models of current conditions, which could either be evidence against the hypothesis of the Long Forest population being isolated by a shift to interglacial conditions, or, more likely, the result of the model being a poor fit to the species' true environmental tolerances.

For either of these hypotheses to be true however, the species needs to have crossed the basalt plains of the Newer Volcanics Province, from which it is completely absent currently and which overlie large areas of the Ordovician sedimentary group (Castlemaine Group) on which the species occurs at Long Forest and the Victorian Goldfields (Figure 2). The clay soils prone to water‐logging that develop on the basalt plain predominately support open, grassy ecosystems (Barlow & Ross, 2001) and the basalt flows partially surround Long Forest aside from the north where the GDR represents exposed parts of the Castlemaine Group. It is possible that it was these volcanic events that isolated the Long Forest population, however, the most recent flows (<0.1 Mya) are to the south of the Long Forest area, with many of the flows that surround Long Forest being closer to 2 Myr old (Heath et al., 2020). This date would seem too old to be the initial cause of the isolation of the Long Forest population given the lack of morphological and genetic differentiation of this population, and the fact that such an old isolation would also infer that the deeper genetic disjunction across the Lower Murray Basin was on the order of several million years. The only possible route of population connection that does not cross basalt flows is across the GDR to the north of Long Forest, which is potentially congruent with a climate driven vicariance event as evidence suggests a less wooded environment on the ranges during the drier, colder glacial climates (Hope, 1994). If *E. behriana* is primarily responding to rainfall and not temperature, it is possible that the species occurred on the GDR during glacial climates and has been outcompeted by taller communities moving upslope in response to increasing rainfall and temperature during an interglacial period. When considering the balance of the evidence from the genetic analyses, SDMs and the known historical environmental change in the region, we favor a hypothesis of climate driven isolation, likely via either the Kilmore Gap or directly across the GDR.

## CONFLICT OF INTEREST

The authors have no conflicts of interest to declare.

## AUTHOR CONTRIBUTION

**Patrick S. Fahey:** Conceptualization (equal); Data curation (lead); Formal analysis (lead); Investigation (lead); Methodology (equal); Project administration (equal); Visualization (lead); Writing‐original draft (lead); Writing‐review & editing (lead). **Rachael M. Fowler:** Formal analysis (supporting); Investigation (supporting); Methodology (equal); Writing‐review & editing (supporting). **Todd G. B. McLay:** Formal analysis (supporting); Investigation (supporting); Methodology (equal); Software (supporting); Writing‐review & editing (supporting). **Frank Udovicic:** Conceptualization (equal); Funding acquisition (lead); Project administration (supporting); Resources (equal); Supervision (supporting); Writing‐original draft (supporting); Writing‐review & editing (supporting). **David J. Cantrill:** Conceptualization (equal); Funding acquisition (equal); Project administration (supporting); Resources (equal); Supervision (supporting); Writing‐original draft (supporting); Writing‐review & editing (supporting). **Michael J. Bayly:** Conceptualization (equal); Funding acquisition (lead); Investigation (supporting); Methodology (equal); Project administration (equal); Resources (lead); Supervision (lead); Visualization (supporting); Writing‐original draft (supporting); Writing‐review & editing (equal).

## Data Availability

Ipyrad parameters tested and individual statistics: Dryad https://doi.org/10.5061/dryad.v9s4mw6sm; and Alignment and SNP dataset used in analyses: Dryad https://doi.org/10.5061/dryad.v9s4mw6sm
